# Hematological profile and risk factors associated with pulmonary tuberculosis patients in Quetta, Pakistan.

**DOI:** 10.12669/pjms.301.4129

**Published:** 2014

**Authors:** Muhammad Shafee, Ferhat Abbas, Muhammad Ashraf, Mohammad Alam Mengal, Niamatullah Kakar, Zafar Ahmad, Fawad Ali

**Affiliations:** 1 Muhammad Shafee, Centre for Advanced Studies in Vaccinology & Biotechnology (CASVAB), University of Balochistan, Quetta, Pakistan.; 2 Ferhat Abbas, Centre for Advanced Studies in Vaccinology & Biotechnology (CASVAB), University of Balochistan, Quetta, Pakistan.; 3 Muhammad Ashraf, Fatima Jinnah Chest & General Hospital, Quetta, Pakistan.; 4 Mohammad Alam Mengal, Centre for Advanced Studies in Vaccinology & Biotechnology (CASVAB), University of Balochistan, Quetta, Pakistan.; 5 Niamatullah Kakar, Fatima Jinnah Chest & General Hospital, Quetta, Pakistan.; 6 Zafar Ahmad, Centre for Advanced Studies in Vaccinology & Biotechnology (CASVAB), University of Balochistan, Quetta, Pakistan.; 7 Fawad Ali, Balochistan University of Information Technology & Management Sciences, Quetta, Pakistan.

**Keywords:** ESR, Hematology, Hemoglobin, Risk factors, Tuberculosis

## Abstract

***Objectives:*** Tuberculosis (TB) is a chronic debilitating infectious disease affecting more than one third of the global population. This study was designed to investigate different peripheral blood parameters and risk factors in TB patients.

***Methods:*** A total of 600 (Male, 238 and Female, 362) aging 20-80 Years patients with clinical signs of prolonged cough, chest pain and fever, were evaluated for peripheral blood parameters using hematology analyzer. All the informations related to the disease were collected from the patients and recorded using predesigned questionnaire.

***Results:*** Erythrocytic Sedimentation Rate (ESR), Hemoglobin (Hb) and lymphocytes were markedly changed in both sexes. Hemoglobin was recorded lower than normal value in 55% and 53% of male and female population respectively. Total leukocyte count was also lower than normal values in 8% and 6% of male and female respectively. Similarly neutropenia was observed in 5% and 8% cases, while neutrophilia was recorded as 60% and 64% in male and female patients respectively. Lymphocytopenia was also observed in 59% and 43% patients in male and female respectively. Illiteracy, smoking habits, overcrowding and living in shared houses were the main associated risk factors contributing in the enhancement of the disease.

***Conclusion:*** The disease was present significantly more in females and was relatively higher in older patients. Different hematological parameters like Erythrocytic sedimentation Rate (ESR), platelets and leukocytes work as hallmark and help the clinicians in early diagnosis of the disease. Malnutrition, smoking tobacco, living in shared houses, illiteracy and poverty were the common risk factors contributing to the dissemination of the tuberculosis in the target area population.

## INTRODUCTION

Tuberculosis (TB) is highly prevalent chronic devastating disease caused by *Mycobacterium tuberculosis* present globally especially in the developing countries including Pakistan.^1^ More than two billion people are currently infected by this disease, of which one in 10 people with tuberculosis (TB) develop active tuberculosis. Each year about 1.8 million mortality occur which equals 4,500 deaths per day. Fourty eight (48%) of them occur in heavily populated Countries, like Pakistan, China, India, Bangladesh, and Indonesia.^2^

*M. tuberculosis *is an acid fast facultative intracellular rod shaped bacterium. It is non-motile, obligate aerobe with long generation time and prefers especially to localize in macrophages.^3^ It affects millions of people each year and is ranked the second leading cause of death from an infectious disease worldwide, after the *Human immunodeficiency virus *(HIV*)*.

Geographically, the burden of TB is highest in Asia and Africa while, India and China together account for almost 40% of the world’s TB cases. Multi drug resistance Tuberculosis (MDR-TB) has been recorded in 3.7% of new and 20% of previously treated cases worldwide. The disease becomes more severe when it is associated with contributory risk factors.^4^

Pakistan is the 6^th^ highest tuberculosis burden country with an estimated incidence of 0.175%). Approximately 3 million people are affected with TB each year and only 8% receive proper treatment while 48000 patients loss their lives in Pakistan.^5^

Reversible peripheral blood abnormalities are commonly associated with pulmonary tuberculosis and these hematological changes act as marker for the diagnosis, prognosis and response to therapy. TB cause profound bone marrow and peripheral blood abnormalities by modulating normal hematopoiesis and the disease become more severe when it is co infected with HIV because of weakened immunity.^6^ In pulmonary tuberculosis many hematological and biochemical abnormalities are common and they are valuable aids to diagnosis.^7^

Balochistan is the largest province of Pakistan accounting for 44% of the total country area. The population is scattered and living in remote areas in a traditional tribal lifestyle. Despite the effective directly observed treatment short course (DOTS) programme in the province, approximately 20,000 new cases of TB were recorded in 2012.^8^ Keeping the above increasing trend in consideration, this study was designed to observe the hematological changes and associated risk factors in smear positive pulmonary tuberculosis patients in the target area.

## METHODS

***Study area: ***The study was conducted at Fatima Jinnah General & Chest Hospital Brewery Road Quetta. The City is located at Latitude: 30°11′13″ N and Longitude: 67°00′45″ E. The provincial capital known as the fruit garden of Balochistan due to the diversity of its plant and animal wildlife. It is the 6th largest city of Pakistan with approximately 2.8 million population.^9^

***Study Design: ***A total of 600 consecutive tuberculosis patients with 20-80 Years age diagnosed by positive bascilloscopy and 30 Healthy control subjects ( n=15 male and n= 15 female each, consisting of 5 subjects from each of three age groups ,20-40,41-60 and 61-80 Years) were recruited for the study. Data regarding gender, age, life style, socioeconomical status and education from all participants were collected through standardized predesigned questinarae to glean information on socio demographic and other possible risk factors characters.

***Sputum collection and slide preparation: ***Sputum samples from all the suspected TB patients (with the history of prolonged coughing, fever and chest pain with more than two weeks) were collected in sterile container and smear microscopy was performed at the provincial reference laboratory for Tuberculosis, Quetta. All the smears were stained with Ziehl Neelsen stain using standard protocol.^10^ Briefly, two drops of sputum were placed onto a clear glass slide, heat dried and stained with 1% solution of carbol fuchsin. The slides were then heated gently for 5 minutes. Then rinsed with water and flooded with acid alcohol. Subsequently were then counter stained with 0.1% methylene blue for 1 minute. All the excess water was drained and slides were air dried after gentle washing and observed under microscope.

***Blood collection and processing:*** About 3-4 ml peripheral venous blood was drawn aseptically with the help of sterile syringe. Two ml was transferred into a tube containing 0.2 ml of 4% Ethylene diamine tetra acetic acid (EDTA) solution and analyzed in the Hematology Analyzer (Sysmex, KX-21) for evaluation of different blood parameters. The remaining 2 ml of the blood were used for Erythrocytic sedimentation rate (ESR) determination. Approximately 02 ml of the anticoagulant added blood was drawn into a Westergen tube up to the mark. The tube was placed in a stand vertically for one hour and the readings were recorded.

***Ethical Information:*** The study was conducted with the prior approval of ethical committee of Institution, University of Balochistan, Quetta. The informed verbal consents were obtained from the patients who participated in this study.

***Statistical Analysis:*** The collected data were subjected to statistical analysis using Chi square test. There was a significant association (P<0.05) among age, gender and different risk factors, like marital status, ethnicity, litracy, employment, crowding index, residence type and socio economical status of the patients.

## RESULTS

***Study Population: ***A total of 600 (Male 238, Female 362) smear positive patients and 30 healthy controls ( 5 from each age group of both sexes) visiting Fatima Jinnah General & Chest Hospital (Provincial TB Reference Laboratory) Brewery Road, Quetta were recruited to evaluate different hematological parameters of patients with tuberculosis and risk factors associated with the transmission of pulmonary tuberculosis. The patients from Four major ethnic groups viz, Pashtoon (41%), Balochi (26%), Sindhi (19%) and other languages (Urdu, Persian and Uzbek) 14% were included in the study.

The age and sex wise analysis of the patients showed that 207 (34.5%) patients (Male 111, Female 96) were from age group of 20-40 Years, 313 (52%) patients (Male 95, Female 218) were from the age group of 41-60 years while 80 (13.5%) patients (Male 32, Female 48) were 61-80 years of age. The mean age of 46.58 (±13.28) years and patients with minimum of 22 Years and maximum of 75 years were recorded.

**Blood parameters** **:**

***Blood Plasma*** **:**

The Erythrocytes Sedimentation rate (ESR) values were recorded under three different categories (ESR values with 15-50, 51-100 and >100mm/hr). More than 50% patients were with ESR 35-50 mm/hr, while 9 cases were exceeded 100 mm/hr, all from female patients.

***Erythrocytes: ***Hemoglobin was observed critically lower than normal values (12-13 g/dl) with 131(55%) and 191 (53%) in male and female patients respectively. Anemia was predominantly evident in 322 (54%) of the total patients. The hematocrit value also revealed lower index than normal in 96 (40%) and 153 (42%) male and female patients respectively.

***Leukocytes: ***The WBC count presented variable features. Total leukocytes count was lower than normal in 19 (8%) and 20 (6%), while lymphocytopenia in 140 (59%) and 157 (43%) in male and female patients respectively. Neutrophilia was observed in 145 (64%) and 232 (60%) while neutropenia in 5 (2.1%) and 8 (2.2%) of the male and female subjects correspondingly.

***Thrombocytes: ***Although Platelets count was found in the normal range in most of the patients, however thrombocytopenia were observed in 36 (15%) and 47 (13%) while thrombocytosis in 29 (12%) and 36 (10%) in male and female patients respectively.

***Risk Factors: ***Malnutrition, poverty, less access to health diagnostic facilities in periphery, illiteracy, smoking, low socioeconomical status, living in shared houses and chronic pulmonary diseases were the main risk factors in patients of the targeted population. No patient was registered with alcoholism. Twenty nine (4.83%) patients were having the history of interaction with tuberculosis patients in the family or in the surroundings. The female population were relatively more contributory to the different risks of acquiring disease as compared with male patients.

## DISCUSSION

Tuberculosis remains a major public health problem in the developing countries, as it is the biggest cause of death in the world from a single infectious disease. This study reveals the hematological profile and associated risk factors to pulmonary tuberculosis in the provincial capital Quetta, Pakistan.

**Table-I:** Characteristics with sociodemographical data of TB Positive patients in Quetta, Pakistan

*Characters*	*Total n=600*
*Age Groups (Years)*	Patients with percentage
20-40	207 (34.5)
41-60	313 (52)
61-80	80 (13.5)
*Gender*	
Male	238 (40)
Female	362 (60)
*Marital status*	
Married	478 (80)
Unmarried	122 (20)
*Ethnicity (Languages spoken)*	
Pashtoon	247 (41)
Others	83 (14 )
Sindhi	116 (19 )
Balochi	154 (26 )
*Education*	
Literate	213 (35.5)
Illiterate	387 (64.5)
*Employment*	
Employed	145 (24)
Unemployed	455 (76)
*Income Per Month (Pak-RS)*	
<20,000	373 (62)
>20,000	227 (38)
*Socioeconomical Status*	
Upper	NIL (00)
Middle	242 (40)
Lower	358 (60)
*Crowding Index*	
>5 person /House	467 (78)
1-5/Person/ House	133 (22)
*History of Tuberculosis in Family or Close Vicinity*	29 (4.83)
* Smoking Habbit*	157 (26)
*Alcoholism*	NIL
*Type of Residence*	
1- Urban	125 (21)
2- Rural	475 (79)

On gender basis the disease was predominantly recorded more in female than male population. Our findings corroborate with Ayaz et al, Baloch et al and Ullah et al,^11^^-^^13^ who also reported more prevalence in female population. This could be attributed to less access by female to hospitals, least health diagnostic facilities at door steps, communal living life style of female in rural settings and illiteracy in female population in our society. A previous study report conducted in Balochistan Pakistan also revealed 1:14 Male to Female ratio of tuberculosis as a matter of great concern as reported by Dogar et al.^14^ Increased rate of progression of tuberculosis might be seen in societies where female remain neglected in term of nutrition especially in strict vegetarians areas. The women also remain neglected in western provinces of Pakistan in term of treatment as they are mostly confined to be household women, maximizing the chances of more contacts with infected carrier.

In this study the disease was equally found in all age groups but was highest (52%) in mature and productive age group of 40-60 Years. This finding was in line with previous study by Sutherland and Fayers, Amin et al and Ullah et al, ^15^^-^^17^ who reported the tuberculosis as a disease of adult productive age group of 20-50 years. Risk of acquiring TB infection increases with age from infancy to early adult life probably, because of increasing number and higher frequency of contacts. It might also be attributed to weakened body immunity in older ages. 

Haematological studies have been conducted by different authors in the past with different results. In our study anaemia (Haemoglobin <12-13 g/dl as stated by Sei^18^, was marked in both the genders due to decreased level of haemoglobin. As 131 (55%) and 191 (53%) male and female patients were with lower index of haemoglobin. In some previous studies conducted by Lombard and Mansvelt and Charles et al, also reported anaemia in most of tuberculosis patients.^7^^,^^19^ This may be attributed to cytokines secreted by macrophages active against tubercle bacilli resulting in decreased erythropoietic production leading to blockage in the reticuloendotheial transfer of iron in the developing RBC. This decrease in haemoglobin might also be due to effects of antituberculosis drugs during the course of treatment. As treatment with chemotherapy goes on, a respectable gradual improvement in the levels of Hb and PCV towards control levels in patients with regular treatments is noticed. This rise in haemoglobin and haematocrit levels can be used as a markers reflecting response to treatment.^20^

ESR is regarded as test of activity in pulmonary tuberculosis. Elevated ESR to different level is one of the indicators of severity of disease and a prognostic tool, was evident in our study. It elevates in those patients with increase in sputum positivity. In earlier studies the elevated ESR is also reported by different scientists in tuberculosis patients. These findings are in agreement with previous studies by Chakraborti et al, Doedhare and Hungund et al.^21^^-^^23^ The WBC also exhibited different abnormal pictures, as lymphocytosis and neutrophilia was seen in most of the cases. This finding is in accordance with former study in which increased numbers of neutrophils and lymphocytes in TB patients were reported in Ibadan, Nigeria.^24^ However our study contradict with another study in India by Hungund et al, who observed much less number of these formed elements in tuberculosis patients.^23^ This increase in lymphocytes and neutrophils may be due to encounter with bacteria in the body resulting in the production of cellular immunity.

Thrombocytopenia and thrombocytosis was also observed in most of the patients in our study, as reported earlier by Hungund et al and Olaniyi and Akeuova.^23^^.^^24^ Thrombocytosis is assumed to be due to increased thrombopoietic factors as an inflammatory response. Varied mechanisms like drugs immune mechanisms, bone marrow fibrosis and hypersplenism have all been implicated as possible causal factors for thrombocytopenia. Interleukin-6 has also been regarded as potent thrombotic factor released by inflamed cells^6^.

The magnitude of TB was high among the poor, displaced, homeless, tobacco smokers, illiterate and lowered socioeconomical status people (Table-I). These factors were also reported in earlier studies Reichman and Hershfield's and Oliva et al.^25^^, ^^26^ None of the patients were found with alcoholism. As this is prohibited in Islam and Muslims don’t use it commonly, even not a single patient confessed to use it but it could be the possible factor if used excessively.

## CONCLUSION

Haematological and biochemical abnormalities in pulmonary tuberculosis are common and may be valuable aids in diagnosis. There was elevated level of ESR in all the patients to substantial level whereas Haemoglobin (Hb) was lower in most of the patients presenting anaemic situation. The WBC exhibited varying degree of alteration with neutropenia and lymphopenia. The platelet count was also lower than normal in most of the patients. Some haematological abnormalities are quite common in patients with pulmonary TB and physicians must maintain a high index of suspicion for diagnosis of pulmonary TB in patients with these abnormalities. However these parameters can be used as indicators in the assessment of response to chemotherapy. In view of the varied haematological abnormalities observed in patients with tuberculosis in patients of this geographical location. We suggest the differential diagnosis of tuberculosis should be entertained in patients with varied haematological disorders and effective awareness programmes should be launched in rural areas to minimize the chances of spread of the disease.
